# Outreach and support in South-London (OASIS) 2001—2020: Twenty years of early detection, prognosis and preventive care for young people at risk of psychosis

**DOI:** 10.1016/j.euroneuro.2020.08.002

**Published:** 2020-10

**Authors:** Paolo Fusar-Poli, Thomas Spencer, Andrea De Micheli, Victoria Curzi, Sunil Nandha, Philip McGuire

**Affiliations:** a Early Psychosis: Interventions and Clinical-detection (EPIC) Lab, Department of Psychosis Studies, Institute of Psychiatry, Psychology & Neuroscience, King's College London, London, UK; b OASIS Service, South London and Maudsley NHS Foundation Trust, London, UK; c Department of Brain and Behavioral Sciences, University of Pavia, Pavia, Italy; d Department of Psychosis Studies, Institute of Psychiatry, Psychology & Neuroscience, King's College London, London, UK

**Keywords:** Psychosis, Schizophrenia, Prevention, ARMS, UHR, CHR

## Abstract

This study aims to describe twenty years of early detection, prognosis and preventive care in the Outreach and Support In South-London (OASIS) mental health service for individuals at Clinical High risk of psychosis (CHR-P). The study presents a comprehensive analysis of the 2001– 2020 activity of the OASIS team encompassing core domains: (i) service characteristics, (ii) detection, (iii) prognosis, (iv) treatment and (v) clinical research. The analyses employed descriptive statistics, population-level data, the epidemiological incidence of psychosis, Kaplan Meier failure functions and Greenwood 95% CIs and Electronic Health Records. OASIS is part of the South London and Maudsley (SLaM) NHS trust, the largest European mental health provider, serving a total urban population of 1,358,646 individuals (population aged 16-35: 454,525). Incidence of psychosis in OASIS's catchment area ranges from 58.3 to 71.9 cases per 100,000 person-years, and it is higher than the national average of 41.5 cases per 100,000 person-year. OASIS is a standalone, NHS-funded, multidisciplinary (team leader, consultant and junior psychiatrists, clinical psychologists, mental health professionals), transitional (for those aged 14-35 years) community mental health service with a yearly caseload of 140 CHR-P individuals. OASIS regularly delivers a comprehensive service promotion outreach to several local community organisations. Referrals to OASIS (2366) are made by numerous agencies; about one-third of the referrals eventually met CHR-P criteria. Overall, 600 CHR-P individuals (55.33% males, mean age 22.63 years, white ethnicity 46.44%) have been under the care of the OASIS service: 80.43% met attenuated psychotic symptoms, 18.06% brief and limited intermittent psychotic symptoms and 1.51% genetic risk and deterioration CHR-P criteria. All CHR-P individuals were offered cognitive behavioural therapy and psychosocial support; medications were used depending on individual needs. The cumulative risk of psychosis at ten years was 0.365 (95%CI 0.302-0.437). At six years follow-up, across two-third of individuals non-transitioning to psychosis, 79.24% still displayed some mental health problem, and only 20.75% achieved a complete clinical remission. Research conducted at OASIS encompassed clinical, prognostic, neurobiological and interventional studies and leveraged local, national and international infrastructures; over the past ten years, OASIS-related research attracted about £ 50 million of grant income, with 5,922 citations in the international databases. Future developments may include broadening OASIS to prevent other serious mental disorders beyond psychosis and fostering translational risk prediction and interventional research. With a twenty-years activity, OASIS’ cutting-edge quality of preventive care, combined with translational research innovations, consolidated the service as a leading reference model for evidence-based prevention of psychosis worldwide.

## 1 Introduction

Preventive interventions in young individuals at Clinical High Risk for Psychosis (CHR-P)(Fusar-Poli, 2017a; Fusar-Poli et al., 2015a) have become a core area of research and clinical care in psychiatry. CHR-P individuals accumulate several risk factors for psychosis(Fusar-Poli et al., 2017e; Oliver et al., 2019; Radua et al., 2018) which trigger attenuated psychotic symptoms(Fusar-Poli et al., 2013a) and functional impairments(Fusar-Poli et al., 2015c). Because of these problems, these individuals seek help(Falkenberg et al., 2015) at specialised CHR-P clinical services(Fusar-Poli et al., 2013b; Fusar-Poli et al., 2019d; Kotlicka-Antczak et al., 2020). Preventive care implemented in these CHR-P services (termed as primary indicated prevention) has the potential to maximise the benefits of early interventions for psychosis(Fusar-Poli et al., 2017b). While the accomplishments and challenges of the CHR-P paradigm have been appraised in recent publications (Fusar-Poli et al., 2020; Salazar de Pablo et al., 2019), there is limited research describing the core characteristics of CHR-P services, which are a core component of the successful delivery of preventive approaches in clinical practice. The Outreach and Support in South-London (OASIS) service is one of the first and largest CHR-P services worldwide. The current study updates an earlier work(Fusar-Poli et al., 2013b) to describe the twenty-years impact of OASIS in this area. This study comprehensively addresses how OASIS has become an established model of care for key components of CHR-P practice: detection, prognosis, treatment (Fusar-Poli et al., 2019e) and research.

## 2 Experimental procedures

The OASIS service was described according to core domains: (i) service characteristics, (ii) detection, (ii) prognosis, (iv) treatment and (v) clinical research. The sample analysed in the current study included all individuals accessing OASIS since its foundation to June 2018, to allow a meaningful follow-up time that represents the typical duration of care under OASIS (i.e. 2 years). Sociodemographic and clinical characteristics of the sample (including missing data), were described with mean and SD for continuous variables, and absolute and relative frequencies for categorical variables. The current (2018) local population was estimated in each borough through the london.gov.uk website. We reported both the total population and the 16-35 age group, to better match the age range of the CHR-P individuals. The local, as well as the national incidence of psychosis for the age range 16-35, was estimated for each borough using PsyMaptic (http://www.psymaptic.org). Follow-up started at the time of acceptance to OASIS and ended when an outcome was recorded, or when the patient dropped out of the follow-up (as documented by the last entry on the local Electronic Health Record, EHR). The cumulative probability of developing a first-episode of psychosis was described through Kaplan Meier(Kaplan and Meier, 1958) failure functions (1-survival)(Kaplan and Meier, 1958) and Greenwood 95% CIs(Greenwood, 1926). We reported the numbers of those at risk and truncate the failure function when less than ten individuals were still at risk. Statistical tests were two-sided and statistical significance was defined as *p*<0.05. All analyses were conducted in STATA 14 (STATA Corp., TX, USA).

## 3 Results

### 3.1 Service characteristics

#### 3.1.1 Catchment area

The OASIS service -established in 2001- is one of the oldest CHR-P services in the UK and worldwide. A previous publication has summarised the achievements of the first ten years (2001-2010) of the service (Fusar-Poli et al., 2013b). OASIS is part of the South London NHS Foundation Trust (SLaM). SLaM is Europe's largest mental health provider, encompassing a population of 1,358,646 individuals (population aged 16-35: 454,525). SLaM includes four boroughs in South-London: Lambeth (total population 334,724; population aged 16-35 133,543 in 2018), Southwark (total population 322,302; population aged 16-35 120,948 in 2018), Lewisham (total population 310,324; population aged 16-35 98,698 in 2018), and Croydon (total population 391,296; population aged 16-35 101,336 in 2018)(Mayor of London, 2018). As CHR-P individuals are by definition help-seeking(Falkenberg et al., 2015), it is not possible to measure their epidemiological incidence in the local catchment area. Incidence of psychosis in SLaM is one of the highest worldwide(Jongsma et al., 2019), presumably in the light of the accumulation of several risk factors for psychosis such as immigration, ethnic minorities and illicit substances misuses(Fusar-Poli et al., 2017e; Oliver et al., 2019; Radua et al., 2018). Specifically, the incidence of psychosis in Lambeth (71.9 cases per 100,000 person-years), Southwark (69.6 cases per 100,000 person-years), Lewisham (71.3 cases per 100,000 person-years) and Croydon (58.3 cases per 100,000 person-years) are all higher than England national average of 41.5 cases per 100,000 person-year. The OASIS service was traditionally established in the SLaM boroughs of Lambeth and Southwark and in 2014-2015 it has expanded in two additional SLaM boroughs of Croydon and Lewisham, becoming a trust-wide service. The OASIS service is currently split across two teams: OASIS Lambeth and Southwark and OASIS Croydon and Lewisham.

#### 3.1.2 Service configuration

OASIS is a standalone, NHS-funded community mental health service which provides early detection, prognostic assessment and preventive treatment for young CHR-P individuals. It is part of the local early intervention pathway, which also includes first-episode of psychosis mental health services: Lambeth Early Onset (LEO), Southwark Treatment for Early Psychosis (STEP), Lewisham Early Intervention Service (LEIS) and Croydon Outreach and Assertive Support Team (COAST). The early intervention pathway also includes a dedicated inpatient unit for individuals experiencing a first-episode of psychosis at Lambeth Hospital (LEO ward). OASIS has close relationships with these first-episode services, accepting or making referrals from/to them and conducting joint assessments. The OASIS Lambeth and Southwark team shares the same building with LEO and STEP, while the OASIS Croydon and Lewisham team is physically co-located with the COAST and LEIS teams. More recently, SLaM has implemented a single point of access, integrated within primary care to facilitate pathways to care, aligning with a youth-friendly soft entry point into mental health care established in other countries (e.g. the headspace model in Australia)(Fusar-Poli, 2019). The single point of access includes a team of mental health professionals, support workers and social workers who can visit young individuals who require mental health support within 24 hours or signpost them to the appropriate SLaM team, such as encompassing OASIS or first-episode services. Furthermore, OASIS represents the first transitional NHS service, taking care of both adolescents and young adults(Fusar-Poli, 2019). Underage individuals remain under the care of the children and adolescent mental health services (CAMHS), but OASIS provides specialised care as detailed below.

#### 3.1.3 Staffing

Staffing configuration across the two OASIS teams, updated to April 2020 is summarised in eFig. 1. The OASIS team in Lambeth and Southwark includes a full-time team leader, two part-time consultant psychiatrists (0.5 and 0.4 full-time), a part-time trainee in psychiatry (0.8 full-time), a full-time assistant psychologist, a full-time clinical psychologist, a part-time clinical psychologist (0.6 full-time) and two full-time allied health professionals (mental health nurse, social worker or occupational therapist); the yearly caseload is of about 80 individuals. The OASIS team in Croydon and Lewisham includes a full-time team leader, two part-time consultant psychiatrists (0.5 and 0.25 full-time), a part-time assistant psychologist (0.6 full-time), two full-time clinical psychologists and two full-time allied health professionals; the yearly caseload is of about 60 individuals. The team is therefore multidisciplinary: medical doctors typically take care of the initial CHR-P assessments, medical reviews and prescription of psychotropic medications, and mental health assessment including compulsory admission to hospital. Allied health professionals operate as keyworkers for the provision of psychosocial, vocational and housing support, housing; assistant and clinical psychologists provide the recommended first-line treatment (cognitive behavioural therapy). All clinicians are also involved in the outreach campaign (see below). The OASIS team has a duty system with a clinician available every day to manage crises and clinical emergencies. The team operates from 9 AM to 5 PM Monday to Friday; the patients access the local accident and emergency services during the nights and weekends. The team leaders coordinate the several members of the team and its operational integration with SLaM services, policies and guidelines.

**Fig. 1:**
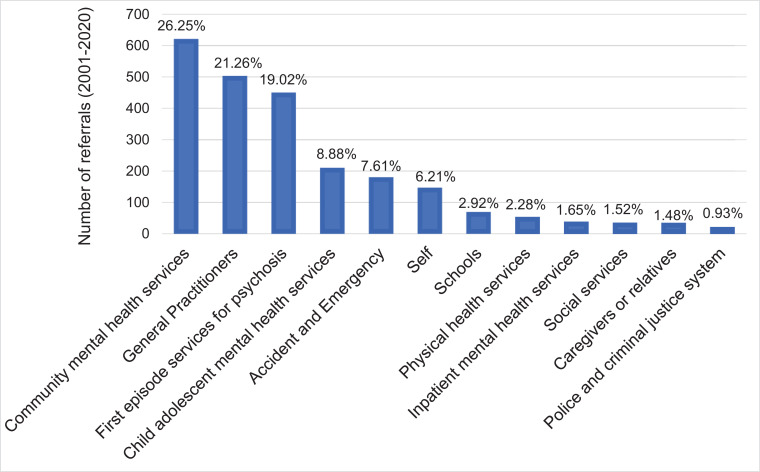
Referrals to OASIS (Lambeth, Southwark, Croydon and Lewisham) from 2001 to 2020, *n*=2366.

### 3.2 Detection

#### 3.2.1 Outreach

The first stage for successfully detecting CHR-P individuals is the running of a comprehensive and ongoing outreach campaign. Outreach conducted at CHR-P services is also essential to enrich the risk of individuals undergoing an assessment for psychosis risk (Fusar-Poli, 2017b; Fusar-Poli et al., 2016e; Fusar-Poli et al., 2016f). OASIS has developed a comprehensive and ongoing outreach programme for raising awareness of risk to psychosis, promoting referrals, and informing/educating other professionals. OASIS regularly delivers service promotion presentations and community engagement visits to several local community organisations including healthcare services such as children and adolescents mental health services, inpatient mental health services (IMHS), general practitioners (GP), perinatal mental health services, sexual health services; housing and employment services; schools and colleges; youth centres; religious or faith centres; charities; wellbeing centres; local businesses (e.g. barber shops, nail bars, betting shops, cafes, pharmacies, theatres). OASIS is particularly active in community engagement projects focused on engaging young people from Black, Asian and minority ethnic backgrounds, asylum-seekers and refugees. This work has enabled OASIS to adopt a sensitive, thoughtful and flexible approach to engaging and working with young people from different cultural backgrounds and has been influential in building trust and developing stronger relationships between OASIS and local communities. In 2019 OASIS’ outreach has been optimised by the creation of a new youth-friendly website (https://www.meandmymind.nhs.uk), co-produced with the OASIS service users group. The latter has been established to advise on service development, tackle the stigma associated with psychosis prevention, and shape the service in response to the needs of young people accessing it, particularly those from different backgrounds and cultures. Further details on the outreach activities of OASIS have been presented in previous publications (Fusar-Poli et al., 2013b; Fusar-Poli et al., 2016e). Overall, OASIS’ outreach strategies have remained stable over recent years, yielding a stable risk enrichment in those undergoing a CHR-P assessment (Fusar-Poli et al., 2017c).

#### 3.2.2 Referrals

**Fig. 2:**
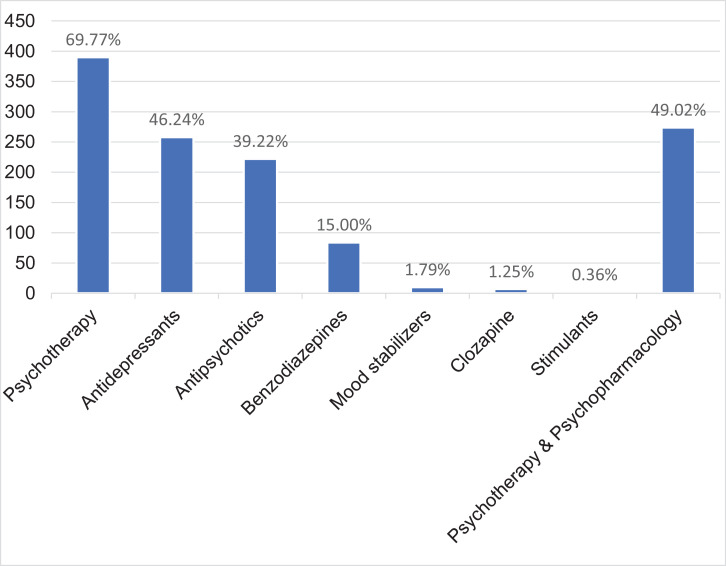
Treatments offered to CHR-P individuals from intake to follow-up. The categories are not mutually exclusive.

Referrals to OASIS typically come from a variety of sources, closely mapping the comprehensive outreach activities implemented. The sources of referral from 2001 to 2020 to OASIS is presented in Fig. 2. Among 2366 referrals, about two-thirds came from CMHS (26.25%%, which include referrals made by the single point of access), general practitioners (21.26%) and first-episode services (19.02%); other frequent sources of referrals were CAMHS (8.88%), accident and emergency departments (7.61%) or self (6.21%). Additional sources of referrals less frequent (<5%) encompassed schools or colleges, physical health services, IMHS, social services, caregivers or relatives, and police and criminal justice system.

### 3.3 Prognosis

#### 3.3.1 Psychometric prognostic assessment

Inclusion criteria at OASIS are: (i) being aged 14-35 (the CHR-P criteria have been mostly validated on this age range) (Fusar-Poli et al., 2020), (ii) having the general practitioner in the borough of Lambeth, Southwark, Croydon or Lewisham, (iii) being help-seeking, and (iv) meeting the CHR-P criteria (determined with the Comprehensive Assessment of At Risk Mental States (CAARMS) 12/2006 (Yung et al., 2005)). The CAARMS criteria include at least one of the following: Attenuated Psychosis Symptoms (APS), Brief and Limited Intermittent Psychotic Symptoms (BLIPS) and Genetic Risk and Deterioration syndrome (GRD) (Fusar-Poli et al., 2016b). At OASIS, the duration of BLIPS is measured continuously on a scale from 1 to 30 days. This has allowed including the standard CAARMS-defined BLPIS as well as extended BLIPS cases (Fusar-Poli et al., 2016a; Fusar-Poli et al., 2017a), which may present with psychotic symptoms that last from 8 to 30 days and then remit in full. This has been introduced because individuals with short-lived psychotic episodes lasting more than 7 but less than 30 days present unmet needs: they are typically felt to be not severe enough under first-episode services and too severe (or over threshold on the CAARMS) for CHR-P services (Fusar-Poli et al., 2019c; Minichino et al., 2018). Because of the high incidence of psychosis in SLaM, BLIPS are relatively frequent in the OASIS service (see below). Furthermore, while substance misuse other than cannabis or alcohol is typically considered an exclusion criterion on the CAARMS, at OASIS this criterion is relaxed, and only individuals with use of multiple illicit substances are screened out a priori. This was again done to meet the sociodemographic characteristics and needs of the local catchment area, which is characterised by frequent use of illicit substances. Comorbid medical conditions, neurodevelopmental disorders and a borderline intelligence quotient (70-85) do not represent a priori exclusion criteria at OASIS but are evaluated on a case by case basis. However, primary diagnoses of medical conditions or neurodevelopmental disorder that are longstanding and severe, or a low intelligence quotient (less than 70) represent exclusion criteria. These features are assessed clinically by the OASIS clinicians. Cognitive functioning is typically assessed as part of research projects, and the results are then fed back to the OASIS service. According to the CAARMS, substance misuse other than cannabis (lifetime cannabis use is reported by 73.6% of OASIS patients, although most of them had discontinued it before clinical presentation (Valmaggia et al., 2014)) and alcohol represent exclusion criteria. Because the CAARMS is transdiagnostic, 70.3% of OASIS patients presented non-psychotic comorbid mental disorders: depressive disorders alone (26%) or in association with anxiety disorders (14%) were the most common disorders (Rutigliano et al., 2016).

The CAARMS assessment also allows estimating the duration of attenuated positive psychotic symptoms measured on the subdomains P1-P4 (Duration of Untreated Attenuated Psychotic Symptoms, DUAPS), and it is typically complemented by the Social and Occupational Functioning Assessment Scale (SOFAS (Rybarczyk, 2011)) and by the Health of the Nation Outcome Scale (HONOS), which is a mandated NHS outcome measures to address the general symptom severity and social functioning across time (Orrell et al., 1999) (12 items that measure rated on a scale ranging from 0 (no problem) to 4 (severe problem)). The presence of comorbid psychiatric conditions is not an a priori exclusion criteria for OASIS and has been detailed in the previous publications (Fusar-Poli et al., 2014). Psychosis onset is ascertained on the CAARMS and further confirmed by ICD-10 diagnostic criteria.

Cases referred to OASIS are first screened by consulting the referral form and interacting with the referrers to check appropriateness for undergoing a CHR-P assessment. The assessment is conducted by expert clinicians who undergo a standardised psychometric training, which has been described elsewhere (Fusar-Poli et al., 2016c). The outcome of the screening and CHR-P assessments are then discussed within the multidisciplinary team on a weekly basis, and decision is reached through consensus. About one-third of referrals is eventually meeting CHR-P criteria, while another third is diagnosed with a frank first-episode of psychosis and therefore referred to the local first-episode services (Fusar-Poli et al., 2013b). Early detection of a first-episode of psychosis by OASIS can further improve outcomes of this disorder (see (Fusar-Poli et al., 2016d)).

#### 3.3.2 Baseline characteristics of the sample

As shown in Table 1, 600 CHR-P individuals (55.33% males) attended the OASIS service from its set up until June 2018 across all SLaM boroughs (mostly Lambeth, [44.09%] and Southwark [31.39%]). At presentation, their mean age was 22.63 years (range 13-36); 79.59% of them were single, 39.22% unemployed and about one-third (35.14%) were students. Half of CHR-P individuals lived with their own family (49.55%); 17.47% lived in supported accommodations (council flats or hostels), and 3.03% were homeless. The proportion of white (46.44%) and non-white (black 32.37%, Asian 7.12%, other 14.07%) ethnicities was similar. The baseline severity of the total CAARMS symptoms was 34.35; baseline functional level was rather low (SOFAS=54.09) and reflected by an average HONOS score of 11.68. The onset of attenuated psychotic symptoms occurred on average about 1.85 years ahead of the CHR-P designation (DUAPS=676.32 days). DUAPS was 202.42 days in the GRD (SD 125.27), 302.03 days in the BLIPS (SD=896.06) and 773.88 days in the APS (SD=1139.69) subgroups. At OASIS, 80.43% of CHR-P individuals met APS criteria, followed by a substantial proportion of BLIPS (18.06%), while GRD cases were rarer (1.51%). The presence of comorbid substance use is frequent and has been detailed in previous publications (Fusar-Poli et al., 2014).

**Table 1:** Baseline characteristics of the sample.

Table X. Clinical and sociodemographic characteristics of the CHR-P sample				
		*N*	*Mean*	*SD*
Age (years)		598	22.63	4.94^a^
CAARMS severity		470	34.35	15.83
	P1 and P2 severity^b^	539	3.89	1.45
	P1 and P2 frequency^b^	529	4.04	1.39
	P3 severity	531	3.14	1.84
	P3 frequency	510	2.73	1.73
	P4 severity	532	1.75	1.52
	P4 frequency	502	2.32	2.04
DUAPS (days)		522	676.32	1105.40
Baseline SOFAS		527	54.09	13.02
HONOS (adjusted total)		379	11.68	6.95
			*Median*	*IQR*

		*N*	*Count*	*%*

Type of CHR-P subgroup^c^		598		
	APS		481	80.43
	BLIPS		108	18.06
	1-7 days		86	14.38
	8-30 days		22	3.67
	GRD		9	1.51

Gender		600		
	Females		268	44.67
	Males		332	55.33
Borough		567		
	Lambeth		250	44.09
	Southwark		178	31.39
	Lewisham		75	13.23
	Croydon		56	9.88
	Homeless		8	1.41
Ethnicity		590		
	White		274	46.44
	Asian		42	7.12
	Black		191	32.37
	Other		83	14.07
Marital status		583		
	Married		24	4.12
	Separated or divorced		13	2.23
	Single		464	79.59
	In a relationship		82	14.07
Employment status		589		
	Employed		151	25.64
	Student		207	35.14
	Unemployed		231	39.22
Accommodation status		561		
	Living with own family		278	49.55
	Owner		7	1.25
	Rental		146	26.02
	Council flat or hostel		98	17.47
	Homeless		17	3.03
	Other		15	2.67

CAARMS: Comprehensive Assessment of At Risk Mental State; SOFAS: Social and Occupational Functioning Assessment Scale; DUAPS: Duration of Untreated Attenuated Psychotic Symptoms; HONOS: Health Of the Nation Outcome Scale; APS: Attenuated Psychotic Symptoms; BLIPS: Brief and Limited Intermittent Psychotic Symptoms; GRD: Genetic Risk and Deterioration syndrome.

a range 13-36.

b Highest scoring across P1 and P2 to harmonise with older versions of the CAARMS.

c 74 individuals met APS+GRD, 36 APS+BLIP, 4 BLIP+GRD and 4 BLIP+APS+GRD criteria

### 3.4 Treatment

#### 3.4.1 Types of preventive treatments

After inclusion, OASIS provides care for up to 2 years. All individuals are offered the first-line recommended treatment to prevent psychosis (NICE, 2014), namely, cognitive behavioural therapy, along with psychosocial support. Its efficacy on preventing psychosis and other outcomes has been reviewed by our group in recent publications (Davies et al., 2018a; Davies et al., 2018b; Fusar-Poli et al., 2019b). As indicated in Fig. 2 and eTables 1–3, about two-thirds (69.77%) of the baseline CHR-P sample is treated with cognitive behavioural therapy; 46.24% received antidepressants, 39.22% antipsychotics, 15% benzodiazepines, 1.74% mood stabilisers, 1.25% clozapine, 0.36% stimulants. In line with our previous study (Fusar-Poli et al., 2015b), about half of the CHR-P individuals (49.02%), psychopharmacological treatments are used conjointly with psychotherapy. The vast majority (80%) of BLIPS individuals, despite being systematically offered cognitive behavioural therapy, did not fully engage with it and did not receive the minimum effective dose; only 3% of BLIPS individuals received the appropriate dose of cognitive behavioural therapy (Fusar-Poli et al., 2019c). The association between cognitive behavioural therapy and antidepressant intervention was found to be associated with a reduced risk of transition to psychosis, as compared with the association between cognitive behavioural therapy and antipsychotic intervention (hazards ratio = 0.129) (Fusar-Poli et al., 2015b). Family therapy and support are also offered on a case by case basis. Finally, those individuals presenting with concurrent cannabis use are offered specific psychoeducation therapy for substance misuse as part of a cannabis clinic that has been implemented in the local early intervention services for psychosis.

#### 3.4.2 Real-world outcomes

**Fig. 3:**
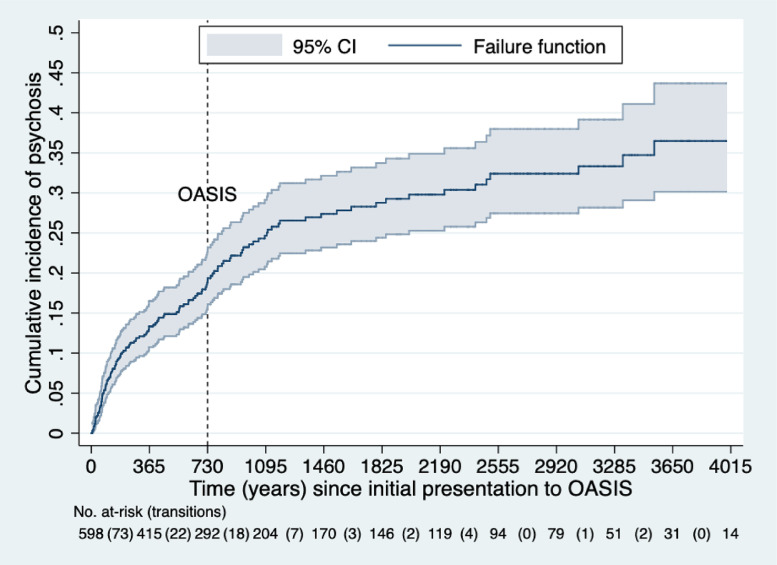
Real-world cumulative risk of transition to psychosis in CHR-P individuals in the long term. The dotted line indicates the maximum duration of care provided by OASIS. Number of at risk individuals for each timepoint are also reported.

The cumulative risk of developing a first ICD-10 psychotic disorder was 0.133 (95%CI 0.107-0.165) at 1 year, 0.191 (95%CI 0.158-0.229) at 2 years, 0.247 (95%CI 0.208-0.291) at 3 years, 0.273 (95%CI 0.231-0.321) at 4 years, 0.288 (95%CI 0.244-0.337) at 5 years, 0.298 (95%CI 0.252-0.349) at 6 years,0.324 (95%CI 0.275-0.379) at 7 and 8 years, 0.333 (95%CI 0.282-0.392) at 9 years, 0.365 (95%CI 0.302-0.437) at 10 and 11 years (Fig. 3). Overall, the cumulative risk for psychosis in the OASIS service has remained substantial, peaking to 40% in the longer term (10 years), and has not declined over the more recent years, as observed elsewhere (Fusar-Poli et al., 2017c).

While in the long term (6 years) about two-third of CHR-P individuals do not develop psychosis, most of those not transitioning (79.24%, corresponding to 56.75% of the baseline sample) still displayed some mental health problem at follow-up: 28.3% of those non-transitioning still reported APS, 45.3% remained functionally impaired and 64.15% present with non-psychotic comorbid mental disorders, with significant overlaps between these outcomes (i.e. persistent APS and non-psychotic comorbid mental disorders co-occurred in 18.86% of non-transitioning cases, persistent APS and functional impairments (i.e. Global Assessment of Functioning score less than 60) co-occurred in 15.09% of non-transitioning cases, non-psychotic comorbid mental disorders and functional impairments co-occurred in 37.73% of non-transitioning cases, persistent APS and non-psychotic comorbid mental disorders and functional impairment all co-occurred in 13.2% of non-transitioning cases) (Rutigliano et al., 2016). Only 20.75% of those non-transitioning (14.9% of the baseline sample) achieved a complete clinical remission at follow-up (Rutigliano et al., 2016). A substantial proportion of the baseline CHR-P sample (56.8%) was affected by at least one comorbid disorder at follow-up: 33.8% mood disorders, 21.6% anxiety disorders, 21.6% personality disorders, 8.1% obsessive-compulsive and related disorders, 6.8% mood and anxiety disorders, 1.4% post-traumatic stress disorder, 1.4% somatoform disorder, 2.7% eating disorders (Rutigliano et al., 2016). However, most of these disorders were carried over from baseline: among CHR-P individuals who presented with some comorbid disorder at baseline, 61.5% had a persistent or recurrent course (Rutigliano et al., 2016). Incident comorbid disorders emerged in 45.4% of baseline CHR-P individuals (Rutigliano et al., 2016), but this proportion is not higher than the control group of individuals assessed but not meeting CHR-P criteria. There is no increased risk of developing incident mental disorders in OASIS individuals compared to help-seeking control groups (Fusar-Poli et al., 2017d).

### 3.5 Clinical research

OASIS is closely linked with the Institute of Psychiatry, Psychology and Neuroscience (IoPPN) at King's College London. This clinical-academic partnership has been developed to improve a research culture within the NHS, with research results feedbacked to OASIS to inform clinical practice, service development, clinical decision making and foster innovation. At the same time, new ideas and questions arising from OASIS clinical practice are turned into researchable projects. The ultimate core of the clinical-academic IoPPN-OASIS partnership is to establish a cutting-edge NHS service where innovations and clinical research are fully integrated, for the benefits of the young people accessing the service.

#### 3.5.1 National and international clinical research infrastructures

OASIS has strong research connection with the Department of Psychosis Studies, which hosts the world's largest group conducting research on psychosis, with over 1300 scientific publications produced in the past five years, and an annual grant income of approximately £60 million in the past year (2018). The Department of Psychosis Studies has received the maximum possible ranking (100% at 4*) for research environment in the 2014 UK Research Excellence Framework; its research impact was evaluated to be 100% "world-leading".

Furthermore, the OASIS team is integrated into the Pan-London Network for Psychosis Prevention (PNP) (Fusar-Poli et al., 2019d), which encompass several CHR-P services across the Greater London (Tower Hamlets Early Detection Service, City & Hackney At Risk Mental State Service, Newham Early Intervention Service, Luton and Bedfordshire Service for the Prevention of Psychosis), serving a total population of 2,318,515 Londoners (830,889; age, 16–35 years), with a yearly recruitment capacity of 220 CHR-P individuals. At the national level, the OASIS is integrated into cutting-edge clinical research National Institute of Health Research (NIHR) Mental Health Translational Research Collaboration Early Psychosis Workstream infrastructure, which includes the best clinical research sites in the UK (Bristol, Cambridge, Maudsley, Oxford, UCL, Exeter, Manchester, University of Newcastle, University of Edinburgh, University of Glasgow, University of Cardiff, Imperial College London, University of Birmingham). At the European level, the OASIS team is part of the European College of Neuropsychopharmacology Prevention of Mental Disorders-Mental Health Promotion Network (ECNP PMD-MHP) (Fusar-Poli et al., 2019a), which includes the best clinical research sites for early intervention and prevention of mental disorders in Europe. Leveraging the urban, national and international infrastructures, over the past two decades, OASIS has played a strategic role leading or contributing to several international research consortia in the field of psychosis prevention -see below.

#### 3.5.2 Clinical research capability

Translational research at OASIS is conducted within The Maudsley Biomedical Research Centre, a major NIHR-funded facility that supports the local clinical infrastructure for hosting mental health research. Neurobiological research at OASIS is typically conducted at the Centre for Neuroimaging Sciences, which allows a world-leading combination of application-oriented brain imaging (perfusion, diffusion, functional and structural imaging and electrophysiology) analysis and clinical expertise, as well as complementary research in imaging physics and analysis. Fluid (blood, plasma) and sample collection at OASIS is done within the Maudsley BioResource for Mental Health. Finally, clinical research and follow-up at OASIS are facilitated by cutting-edge digital EHR infrastructure that is available in SLaM (Stewart et al., 2009). SLaM is paper-free, and all clinical notes are recorded in an HER. SLaM was awarded Global Digital Exemplar status by NHS England in 2017.

#### 3.5.3 Clinical research outputs

Over the past ten years, clinical research programmes led by OASIS (detailed in Table 2) have been substantial, attracting about £ 50 million of grant income. These programmes encompass the study of long term outcomes in CHR-P individuals through machine learning methods, service development (e.g. OASIS in prison service) studies and the development of digital technologies (smartphone apps, EHR screening) to measure CHR-P endophenotypes and outcomes. Furthermore, extensive neurobiological research at OASIS has investigated normative brain charting and trajectories of brain changes for predicting outcomes, the development of neurobiological-based prediction models, the discovery and validation of biomarkers, the international standardisation and harmonisation of neurobiological assessments, and brain-genetic interactions. Neurochemical studies at OASIS have elucidated the role of dopamine, glutamate, GABA, and their interactions during the onset of psychosis in CHR-P individuals. Finally, clinical research at OASIS has strongly focused on experimental medicine and translational discoveries, with several research programmes undergoing to test the efficacy of promising compounds (e.g. cannabidiol, oxytocin) or psychological interventions (e.g. individual placement and support).

**Table 2:** OASIS research programme.

	Funder	Amount	Period
Developing Mobile Digital Technologies to Measure Stress-Biomarker Signatures Across Psychotic Illness Stages	BBRF	GPB 50,000	2020-2022
Normative brain charting for predicting and stratifying psychosis	WT	GPB 494,930	2019-2021
Using smartphone-based personal sensing to understand and predict risk of psychotic relapse at the individual level	MRC	GBP 754,342	2019-2023
Education and Employment focused Individual Placement and Support (IPS) within Early Detection for Psychosis services	Maudsley Charity	GBP 49,653	2019-2020
PSYSCAN: Translating neuroimaging findings from research into clinical practice	EC	Eur 6,000,000	2014-2020
STEP: Stratification & Treatment in Early Psychosis	WT	GBP 15,000,000	2020-2025
HARMONY: Harmonization of At Risk Multisite Observational Networks for Youth	NIMH	US Dollars 750,000	2015-2020
CANTOP-RCT: CANnabidiol as a Treatment fOr Psychosis clinical high risk state- a Randomised Clinical Trial	NIHR	GBP 1,854,492	2019-2024
Linking electronic health records with passive smartphone activity data to predict outcomes in psychotic disorders	MRC	GBP 326,858	2018-2021
Extending the benefits of primary indicated prevention to improve outcomes of Psychosis	MRC	GBP 80,000	2017-2019
NIHR Biomedical Research Centre. Funding for Financial Years 2017/18, 2018/19	NIHR	GBP 20,000,000	2017-2022
Stress and GABA in the pathogenesis of psychosis	WT	GBP 1,089,387	2017-2022
Using smartphone technologies to investigate the effects of the physical and interpersonal environment on coping strategies in early psychosis	BBRF	GBP 44,324	2017-2020
Biological markers of stress and inflammation across clinical stages of schizophrenia: from early at risk states to chronic illness	WT	GBP 250,000	2015-2019
Social context and the development, persistence and outcomes of psychotic symptoms in the general population	WT	GBP 224,765	2013-2017
Neural and behavioural correlates of emotional dysfunction in individuals at risk of schizophrenia	BBRF	GBP 19,000	2014-2016
Is there a change in dopaminergic function with the onset of psychosis?	BBRF	GBP 25,986	2013-2015
Evaluation of cannabidiol as a treatment for the early phase of psychosis	WT	GBP 99,221	2013-2014
Predicting clinical and functional outcomes in psychosis using machine learning	MRC	GBP 174,823	2013-2016
Cannabidiol as a novel therapeutic agent for individuals at ultra-high risk of psychosis: an experimental medicine approach	MRC	GBP 376,857	2013-2015
Trajectory of Brain Structure and Function before and after the Onset of Psychosis: a Longitudinal Multicentre Study	MRC	GBP 1,227,953	2012-2018
OASIS Prison In-reach Team. A service for prisoners at high risk of developing psychosis	Maudsley Charity	GBP 220,127	2012-2014
Neurobiological factors underlying the onset of psychosis	WT	GBP 1,054,325	2011-2017
Structural disorganisation in psychosis and its functional consequences	WT	GBP 180,339	2011-2014
OASIS Prison In-reach Team. A service for prisoners at high risk of developing psychosis	G&T	GBP 376,500	2011-2014
The long term outcome of early detection for psychosis.	G&T	GBP 98,000	2010-2013
Structural brain correlates of an operationally defined High Risk Phenotype for Schizophrenia: a population-based study	MRC	GBP 329,398	2010-2014
European Network of National Schizophrenia Studying Gene-Environment Interaction - Work Package 5: Prodrome	EC	GBP 457,211	2010-2015
Relationship between Dopamine & Glutamate Dysfunction in Schizophrenia	BBRF	GBP 63,666	2010-2013

BBRF: Brain and Behavior Research Foundation; MRC, Medical Research Council; WT, Wellcome Trust; NIHR: National Institute of Health Research; NIMH: National Institute of Mental Health; EC: European Commission; G&T: Guy's and St Thomas' Charity.

**Fig. 4:**
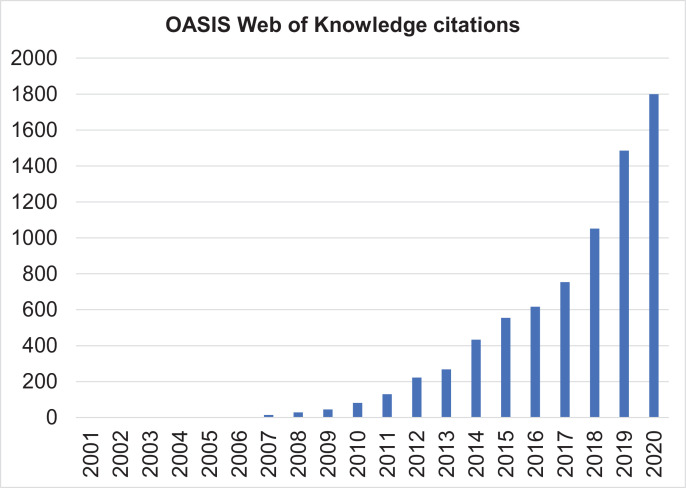
Web of Knowledge citations (using the search terms “OASIS” and “psychosis”) relating to the OASIS service (*n*=5,922, up to March 19^th^, 2020, the number of publications in 2020 is projected).

The scientific publications associated with these research programmes are numerous, to the point that it is not possible to track all of them. As indicated in Fig. 4, OASIS-related scientific publications increased exponentially, reflecting the exponential impact of this service on the scientific community. In 2019, OASIS-related citations cumulated to 5,922 (up to March 19^th^, 2020). As illustrated in Fig. 4, the scientific impact of OASIS is also multidisciplinary extending from psychiatry to neurosciences, clinical psychology, clinical neurology, pharmacology, general medicine, neuroimaging, behavioural sciences, radiology, biochemistry and molecular biology and endocrinology.

#### 3.5.4 The future of OASIS

Over the coming decades, OASIS service will continue to offer evidence-based, innovative preventive care and conduct cutting-edge research in young people with emerging psychosis. However, it is foreseeable that its activity will be broadened to encompass prevention of other severe mental disorders such as bipolar disorders and depressive disorders, to better align with ongoing mental health reforms in this area (Fusar-Poli, 2019). OASIS has recently championed a new psychometric instrument for the detection of individuals at risk of bipolar disorders (the Structured Interview for Bipolar At risk States (Fusar-Poli et al., 2018)), and further applications are undergoing. Furthermore, it is expected that drug discovery and prediction modelling will become two mainstream areas of research at OASIS to fill the current gap of knowledge and ultimately advance clinical care for vulnerable individuals (Fusar-Poli et al., 2020).

## 4 Conclusions

With twenty years of activity, the OASIS service has consolidated its impact as one of the most established CHR-Ps services in the UK and worldwide. OASIS’ cutting-edge quality of preventive care, combined with its translational research innovations, provide a successful template and reference service model for the prevention of psychosis worldwide.

## Role of the funding source

This study was supported by the King's College London Confidence in Concept award from the Medical Research Council (MRC) (MC_PC_16048) to PFP. These funding bodies had no role in the design of the study, collection, and analyses.

## Availability of data

There is no ethical permission for data sharing.

## Ethical standards

The authors assert that all procedures contributing to this work comply with the ethical standards of the relevant national and institutional committees on human experimentation and with the Helsinki Declaration 1975, as revised in 2008.

## Author contributors

PFP conceived the study, conducted the analyses and drafted the manuscript. ADM obtained the data. TS, ADM, VC, SN and PM revised the manuscript and provided a substantial conceptual contribution. All authors proofread and approved the final draft of the manuscript.

## Conflict of interest

The authors declare that the research was conducted in the absence of any commercial or financial relationships that could be construed as a potential conflict of interest. General sources of potential conflict of interest, considered unrelated to this work include the following: PFP has been a consultant to or has received honoraria or research grants from Lundbeck, Angelini, Menarini and Boehringer Ingelheim.

## Appendix Supplementary materials

Image, application 1

## References

[bib0001] Davies C., Cipriani A., Ioannidis J.P.A., Radua J., Stahl D., Provenzani U., McGuire P., Fusar-Poli P. (2018). Lack of evidence to favor specific preventive interventions in psychosis: a network meta-analysis. World Psychiatry.

[bib0002] Davies C., Radua J., Cipriani A., Stahl D., Provenzani U., McGuire P., Fusar-Poli P. (2018). Efficacy and acceptability of interventions for attenuated positive psychotic symptoms in individuals at clinical high risk of psychosis: a network meta-analysis. Front. Psychiatry.

[bib0003] Falkenberg I., Valmaggia L., Byrnes M., Frascarelli M., Jones C., Rocchetti M., Straube B., Badger S., McGuire P., Fusar-Poli P. (2015). Why are help-seeking subjects at ultra-high risk for psychosis help-seeking?. Psychiatry Res..

[bib0004] Fusar-Poli P. (2017). The clinical high-risk state for psychosis (CHR-P), version II. Schizophr. Bull..

[bib0005] Fusar-Poli P. (2017). Why ultra high risk criteria for psychosis prediction do not work well outside clinical samples and what to do about it. World Psychiatry.

[bib0006] Fusar-Poli P. (2019). Integrated mental health services for the developmental period (0 to 25 years): a critical review of the evidence. Front. Psychiatry.

[bib0007] Fusar-Poli P., Bauer M., Borgwardt S., Bechdolf A., Correll C.U., Do K.Q., Domschke K., Galderisi S., Kessing L.V., Koutsouleris N., Krebs M.O., Lennox B., McGuire P., Meyer-Lindenberg A., Millan M.J., Nieman D., Pfennig A., Sand M., Whenert A., van Amelsvoort T., Arango C. (2019). European college of neuropsychopharmacology network on the prevention of mental disorders and mental health promotion (ECNP PMD-MHP). Eur. Neuropsychopharmacol..

[bib0008] Fusar-Poli P., Borgwardt S., Bechdolf A., Addington J., Riecher-Rossler A., Schultze-Lutter F., Keshavan M., Wood S., Ruhrmann S., Seidman L.J., Valmaggia L., Cannon T., Velthorst E., De Haan L., Cornblatt B., Bonoldi I., Birchwood M., McGlashan T., Carpenter W., McGorry P., Klosterkotter J., McGuire P., Yung A. (2013). The psychosis high-risk state: a comprehensive state-of-the-art review. JAMA Psychiatry.

[bib0009] Fusar-Poli P., Byrne M., Badger S., Valmaggia L.R., McGuire P.K. (2013). Outreach and support in south London (OASIS), 2001-2011: ten years of early diagnosis and treatment for young individuals at high clinical risk for psychosis. Eur. Psychiatry.

[bib0010] Fusar-Poli P., Cappucciati M., Bonoldi I., Hui L.M., Rutigliano G., Stahl D.R., Borgwardt S., Politi P., Mishara A.L., Lawrie S.M., Carpenter W.T., McGuire P.K. (2016). Prognosis of brief psychotic episodes: a meta-analysis. JAMA Psychiatry.

[bib0011] Fusar-Poli P., Cappucciati M., Borgwardt S., Woods S.W., Addington J., Nelson B., Nieman D.H., Stahl D.R., Rutigliano G., Riecher-Roessler A., Simon A.E., Mizuno M., Lee T.Y., Kwon J.S., Lam M.M.L., Perez J., Keri S., Amminger P., Metzler S., Kawohl W., Roessler W., Lee J., Labad J., Ziermans T., An S.K., Liu C.-C., Woodberry K.A., Braham A., Corcoran C., McGorry P., Yung A.R., McGuire P.K. (2016). Heterogeneity of psychosis risk within individuals at clinical high risk a meta-analytical stratification. JAMA Psychiatry.

[bib0012] Fusar-Poli P., Cappucciati M., De Micheli A., Rutigliano G., Bonoldi I., Tognin S., Ramella-Cravaro V., Castagnini A., McGuire P. (2017). Diagnostic and prognostic significance of brief limited intermittent psychotic symptoms (BLIPS) in individuals at ultra high risk. Schizophr. Bull..

[bib0013] Fusar-Poli P., Cappucciati M., Rutigliano G., Lee T.Y., Beverly Q., Bonoldi I., Lelli J., Kaar S.J., Gago E., Rocchetti M., Patel R., Bhavsar V., Tognin S., Badger S., Calem M., Lim K., Kwon J.S., Perez J., McGuire P. (2016). Towards a standard psychometric diagnostic interview for subjects at ultra high risk of psychosis: CAARMS versus SIPS. Psychiatry J..

[bib0014] Fusar-Poli P., Cappucciati M., Rutigliano G., Schultze-Lutter F., Bonoldi I., Borgwardt S., Riecher-Rossler A., Addington J., Perkins D., Woods S.W., McGlashan T.H., Lee J., Klosterkotter J., Yung A.R., McGuire P. (2015). At risk or not at risk? A meta-analysis of the prognostic accuracy of psychometric interviews for psychosis prediction. World Psychiatry.

[bib0015] Fusar-Poli P., Davies C., Solmi M., Brondino M., De Micheli A., Kotlicka-Antczak M., Shin J.I., Radua J. (2019). Preventive treatments for psychosis: umbrella reivew (just the evidence). Front. Psychiatry.

[bib0016] Fusar-Poli P., De Micheli A., Chalambrides M., Singh A., Augusto C., McGuire P. (2019). Unmet needs for treatment in 102 individuals with brief and limited intermittent psychotic symptoms (BLIPS): implications for current clinical recommendations. Epidemiol. Psychiatr. Sci..

[bib0017] Fusar-Poli P., De Micheli A., Rocchetti M., Cappucciati M., Ramella-Cravaro V., Rutigliano G., Bonoldi I., McGuire P., Falkenberg I. (2018). Semistructured interview for bipolar at risk states (SIBARS). Psychiatry Res..

[bib0018] Fusar-Poli P., Diaz-Caneja C.M., Patel R., Valmaggia L., Byrne M., Garety P., Shetty H., Broadbent M., Stewart R., McGuire P. (2016). Services for people at high risk improve outcomes in patients with first episode psychosis. Acta Psychiatr. Scand..

[bib0019] Fusar-Poli P., Estrade A., Spencer T.J., Gupta S., Murguia-Asensio S., Eranti S., Wilding K., Andlauer O., Buhagiar J., Smith M., Fitzell S., Sear V., Ademan A., De Micheli A., McGuire P. (2019). Pan-London network for psychosis-prevention (PNP). Front. Psychiatry.

[bib0020] Fusar-Poli P., Frascarelli M., Valmaggia L., Byrne M., Stahl D., Rocchetti M., Codjoe L., Weinberg L., Tognin S., Xenaki L., McGuire P. (2015). Antidepressant, antipsychotic and psychological interventions in subjects at high clinical risk for psychosis: OASIS 6-year naturalistic study. Psychol. Med..

[bib0021] Fusar-Poli P., McGorry P.D., Kane J.M. (2017). Improving outcomes of first-episode psychosis: an overview. World Psychiatry.

[bib0022] Fusar-Poli P., Nelson B., Valmaggia L., Yung A.R., McGuire P.K. (2014). Comorbid depressive and anxiety disorders in 509 individuals with an at-risk mental state: impact on psychopathology and transition to psychosis. Schizophr. Bull..

[bib0023] Fusar-Poli P., Palombini E., Davies C., Oliver D., Bonoldi I., Ramella-Cravaro V., McGuire P. (2017). Why transition risk to psychosis is not declining at the OASIS ultra high risk service: The hidden role of stable pretest risk enrichment. Schizophr. Res..

[bib0024] Fusar-Poli P., Rocchetti M., Sardella A., Avila A., Brandizzi M., Caverzasi E., Politi P., Ruhrmann S., McGuire P. (2015). Disorder, not just state of risk: meta-analysis of functioning and quality of life in people at high risk of psychosis. Br. J. Psychiatry.

[bib0025] Fusar-Poli P., Rutigliano G., Stahl D., Davies C., De Micheli A., Ramella-Cravaro V., Bonoldi I., McGuire P. (2017). Long-term validity of the At Risk Mental State (ARMS) for predicting psychotic and non-psychotic mental disorders. Eur. Psychiatry.

[bib0026] Fusar-Poli P., Rutigliano G., Stahl D., Schmidt A., Ramella-Cravaro V., Shetty H., McGuire P. (2016). Deconstructing pretest risk enrichment to optimize prediction of psychosis in individuals at clinical high risk. JAMA Psychiatry.

[bib0027] Fusar-Poli P., Salazar de Pablo G., Correll C., Meyer-Lindenberg A., Millan M., Borgwardt S., Galderisi S., Bechdolf A., Pfenning A., Kessing L., van Amelsvoort T., Nieman D., Domschke K., Krebs M.-O., Koutsouleris M., McGuire P., Arango C. (2020). Prevention of psychosis: advances in detection, prognosis and intervention. JAMA Psychiatry.

[bib0028] Fusar-Poli P., Schultze-Lutter F., Addington J. (2016). Intensive community outreach for those at ultra high risk of psychosis: dilution, not solution. Lancet Psychiatry.

[bib0029] Fusar-Poli P., Sullivan S., Shah J., Uhlhaas P. (2019). Improving the detection of individuals at clinical risk for psychosis in the community, primary and secondary care: an integrated evidence-based approach. Front. Psychiatry.

[bib0030] Fusar-Poli P., Tantardini M., De Simone S., Ramella-Cravaro V., Oliver D., Kingdon J., Kotlicka-Antczak M., Valmaggia L., Lee J., Millan M.J., Galderisi S., Balottin U., Ricca V., McGuire P. (2017). Deconstructing vulnerability for psychosis: Meta-analysis of environmental risk factors for psychosis in subjects at ultra high-risk. Eur. Psychiatry.

[bib0031] Greenwood M. (1926). A Report on the Natural Duration of Cancer. Issue 33 of Reports on public health and medical subjects.

[bib0032] Jongsma H.E., Turner C., Kirkbride J.B., Jones P.B. (2019). International incidence of psychotic disorders, 2002-17: a systematic review and meta-analysis. Lancet Public Health.

[bib0033] Kaplan E., Meier P. (1958). Nonparametric estimation from incomplete observations. J. Amer. Statist. Assn..

[bib0034] Kotlicka-Antczak M., Podgorski M., Oliver D., Maric N.P., Valmaggia L., Fusar-Poli P. (2020). Worldwide implementation of clinical services for the prevention of psychosis: The IEPA early intervention in mental health survey. Early Interv. Psychiatry.

[bib0035] Mayor of London (2018). London Data Store. https://data.london.gov.uk.

[bib0036] Minichino A., Rutigliano G., Merlino S., Davies C., Oliver D., De Micheli A., Patel R., McGuire P., Fusar-Poli P. (2018). Unmet needs in patients with brief psychotic disorders: too ill for clinical high risk services and not enough ill for first episode services. Eur. Psychiatry.

[bib0037] NICE (2014). Psychosis and Schizophrenia in Adults: Prevention and Management. https://www.nice.org.uk/guidance/cg178.

[bib0038] Oliver D., Reilly T., Baccaredda Boy O., Petros N., Davies C., Borgwardt S., McGuire P., Fusar-Poli P. (2019). What causes the onset of psychosis in individuals at clinical high risk? A meta-analysis of risk and protective factors. Schizophr. Bull..

[bib0039] Orrell M., Yard P., Handysides J., Schapira R. (1999). Validity and reliability of the Health of the Nation Outcome Scales in psychiatric patients in the community. Br. J. Psychiatry.

[bib0040] Radua J., Ramella-Cravaro V., Ioannidis J.P.A., Reichenberg A., Phiphopthatsanee N., Amir T., Yenn Thoo H., Oliver D., Davies C., Morgan C., McGuire P., Murray R.M., Fusar-Poli P. (2018). What causes psychosis? An umbrella review of risk and protective factors. World Psychiatry.

[bib0041] Rutigliano G., Valmaggia L., Landi P., Frascarelli M., Cappucciati M., Sear V., Rocchetti M., De Micheli A., Jones C., Palombini E., McGuire P., Fusar-Poli P. (2016). Persistence or recurrence of non-psychotic comorbid mental disorders associated with 6-year poor functional outcomes in patients at ultra high risk for psychosis. J. Affect. Disord..

[bib0042] Rybarczyk B., Kreutzer J.S., DeLuca J., Caplan B. (2011). Social and Occupational Functioning Assessment Scale (SOFAS). Encyclopedia of Clinical Neuropsychology.

[bib0043] Salazar de Pablo G., Catalan A., Fusar-Poli P. (2019). Clinical validity of DSM-5 attenuated psychosis syndrome: advances in diagnosis, prognosis, and treatment. JAMA Psychiatry.

[bib0044] Stewart R., Soremekun M., Perera G., Broadbent M., Callard F., Denis M., Hotopf M., Thornicroft G., Lovestone S. (2009). The South London and Maudsley NHS Foundation Trust Biomedical Research Centre (SLAM BRC) case register: development and descriptive data. BMC Psychiatry.

[bib0045] Valmaggia L.R., Day F.L., Jones C., Bissoli S., Pugh C., Hall D., Bhattacharyya S., Howes O., Stone J., Fusar-Poli P., Byrne M., McGuire P.K. (2014). Cannabis use and transition to psychosis in people at ultra-high risk. Psychol. Med..

[bib0046] Yung A., Yuen H., McGorry P., Phillips L., Kelly D., Dell'Olio M., Francey S., Cosgrave E., Killackey E., Stanford C., Godfrey K., Buckby J. (2005). Mapping the onset of psychosis: the comprehensive assessment of At-Risk Mental States. ANZJP.

