# Supplementary material for: Outreach and support in South-London (OASIS) 2001—2020: Twenty years of early detection, prognosis and preventive care for young people at risk of psychosis
Source: Eur Neuropsychopharmacol. 2020 Oct;39:111–22. doi: 10.1016/j.euroneuro.2020.08.002 (PMC7540251; doi:10.1016/j.euroneuro.2020.08.002)
Supplement: Supplementary file 1 [file mmc1.docx]

**eFigure 1** Staffing structure of OASIS service (up to April 2020) across South London And Maudsley (SLaM) NHS Foundation Trust: OASIS Lambeth and Southwark and OASIS Croydon and Lewisham.

**eTable 1.** Types of first treatment with antipsychotic molecules prescribed in CHR-P individuals and chlorpromazine equivalents.

| **First Antipsychotic molecule** | | | **Chlorpromazine equivalent** | | |  |
| --- | --- | --- | --- | --- | --- | --- |
| ***Name*** | ***Counts*** | ***Relative frequency*** | ***Counts*** | ***Mean (mg)*** | ***SD*** | |
| Quetiapine | 83 | 38.25 | 80 | 194.38 | 23.12 | |
| Olanzapine | 40 | 18.43 | 39 | 156.41 | 11.64 | |
| Risperidone | 41 | 18.89 | 41 | 99.39 | 8.7 | |
| Aripiprazole | 32 | 14.75 | 31 | 137.09 | 16.39 | |
| Amisulpride | 8 | 3.69 | na | na | na | |
| Promethazine | 13 | 5.99 | na | na | na | |

Na, not available.

**eTable 2**. Types of first treatment with benzodiazepine molecules prescribed in CHR-P individuals.

| **First benzodiazepine molecule** | | |
| --- | --- | --- |
| ***Name*** | ***Counts*** | ***Relative frequency*** |
| Clonazepam | 31 | 36.05 |
| Lorazepam | 4 | 4.65 |
| Diazepam | 17 | 19.77 |
| Temazepam | 2 | 2.33 |
| Chlordiazepoxide | 1 | 1.16 |
| Zopiclone | 29 | 33.72 |
| Clonazepam and Zopiclone | 2 | 2.33 |

**eTable 3**. Types of first treatment with other psychotropic molecules (excluding antipsychotics and benzodiazepines) prescribed in CHR-P individuals.

| **First treatment with molecules other than antipsychotics or benzodiazepines** | | | |
| --- | --- | --- | --- |
| ***Name*** | ***Counts*** | ***Relative frequency*** |  |
| Sertraline | 84 | 31 |  |
| Citalopram | 83 | 30.63 |  |
| Escitalopram | 5 | 1.85 |  |
| Fluoxetine | 37 | 13.65 |  |
| Paroxetine | 7 | 2.58 |  |
| Mirtazapine | 36 | 13.28 |  |
| Venlafaxine | 2 | 0.74 |  |
| Trazodone | 1 | 0.37 |  |
| Amitriptyline | 2 | 0.74 |  |
| Reboxetine | 1 | 0.37 |  |
| Sodium valproate | 7 | 2.58 |  |
| Lithium | 2 | 0.74 |  |
| Metylphenidate | 2 | 0.74 |  |
| Citalopram and Lamotrigine | 1 | 0.37 |  |
| Sertraline and Mirtazapine and Amitriptyline | 1 | 0.37 |  |
